# Factors affecting the incidence of pulmonary tuberculosis based on the GTWR model in China, 2004–2021

**DOI:** 10.1017/S0950268824000335

**Published:** 2024-02-29

**Authors:** Hairu Yu, Jiao Yang, Yexin Yan, Hui Zhang, Qiuyuan Chen, Liang Sun

**Affiliations:** Department of Social Medicine and Health Service Management, College of Public Health, Zhengzhou University, Zhengzhou, the People’s Republic of China

**Keywords:** geographically and temporally weighted regression model, influencing factors, pulmonary tuberculosis

## Abstract

Contra-posing panel data on the incidence of pulmonary tuberculosis (PTB) at the provincial level in China through the years of 2004–2021 and introducing a geographically and temporally weighted regression (GTWR) model were used to explore the effect of various factors on the incidence of PTB from the perspective of spatial heterogeneity. The principal component analysis (PCA) was used to extract the main information from twenty-two indexes under six macro-factors. The main influencing factors were determined by the Spearman correlation and multi-collinearity tests. After fitting different models, the GTWR model was used to analyse and obtain the distribution changes of regression coefficients. Six macro-factors and incidence of PTB were both correlated, and there was no collinearity between the variables. The fitting effect of the GTWR model was better than ordinary least-squares (OLS) and geographically weighted regression (GWR) models. The incidence of PTB in China was mainly affected by six macro-factors, namely medicine and health, transportation, environment, economy, disease, and educational quality. The influence degree showed an unbalanced trend in the spatial and temporal distribution.

## Introduction

According to the Global Tuberculosis Report 2022 [1] released by the World Health Organization (WHO), the number of 10.6 million new tuberculosis (TB) cases occurred worldwide in 2021, and China remained one of the countries with the highest burden of TB. In 2021, China had the third highest number of TB cases after India and Indonesia. The incidence of TB was affected by many factors, such as society–economy, population, climate, transportation, and other factors, which makes TB a multifaceted and complex public health problem [2, 3]. Existing studies analysed and explored the influencing factors of TB onset in China [4, 5]. However, most of the studies used ordinary least-squares (OLS) and geographically weighted regression (GWR) models to analyse influencing factors and did not consider the time and space dimensions, which cannot accurately reflect the spatial–temporal heterogeneity of pulmonary tuberculosis (PTB) onset and affecting factors in China. The geographically and temporally weighted regression (GTWR) model was used to study the spatial–temporal heterogeneity of various diseases [6–8]. Therefore, to provide policies and measures for the regional prevention and treatment of PTB, we used the latest data of the national PTB incidence rate through the years of 2004–2021, as well as twenty-two categories of indicators under the six macro-influencing factors, and analysed the influencing factors of the PTB incidence based on the GTWR model so as to provide policies and measures for the regional prevention and treatment of PTB.

## Methods

### Research data

Data on PTB incidence in China through the years of 2004–2018 were collected from the Data Center of Public Health Science. And the data through the years of 2019–2021 were collected from the China Health Statistics Yearbook. The influencing factors of medical and health, transportation, environment, economy, disease, and educational quality on the incidence of PTB in China were comprehensively considered. A total of twenty-two indicators under six macros (Table 1) were collected from 31 provinces, autonomous regions, and municipalities (excluding Hong Kong, Macao, and Taiwan) through the years of 2004–2021. The vector map of China’s provincial administrative divisions was downloaded by the National Center for Basic Geographic Information System.

**Table 1.** Measurement indicators of macro-influencing factors

Macro-influencing factor	Measure index
Medical and health care	Number of health workers (10 000)
	Number of beds in medical and health institutions (10 000)
	Number of medical and health institutions (per)
	Local expenditure on medical and health care (100 million yuan)
Transportation	Passenger turnover (100 million person–kilometres)
	Automobile ownership in highway operation (10 000 units)
	Highway mileage (ten thousand kilometres)
	Passenger volume (10 000)
	Civil automobile ownership (10 000 units)
Environment	SO_2_ emissions (10 000 tons)
	Smoke (powder) dust discharge (10 000 tons)
	Ammonia nitrogen emissions (10 000 tons)
	Chemical oxygen demand emissions (10 000 tons)
Economy	Per capita disposable income of all residents (yuan)
	PGDP (yuan)
	Per capita consumption expenditure of all households (yuan)
	Consumer Price Index (last year =100)
Disease	AIDS incidence rate (per 10 000)
Educational quality	Number of regular institutions of higher learning (institutions)
	Total number of staff and staff in regular institutions of higher learning (10 000)
	Average number of students in institutions of higher learning per 100000 population
	Educational expenditure (10 000 yuan)

Abbreviations: AIDS, acquired immune deficiency syndrome; PGDP, per capital gross domestic product; SO_2_, sulphur dioxide.

### Principal component analysis

Principal component analysis (PCA) was a multivariate statistical method that converted multiple indicators under six macro-factors into comprehensive indicators with little loss of information by using the method of dimensional reduction. Kaiser–Meyer–Olkin (*KMO)* and Bartlett tests were used in this study. And all macro-influencing factors were standardized and normalized.

### Correlation analysis and collinearity diagnosis

R 4.2.0 was used to conduct the Spearman correlation test between each macro-influencing factor and the incidence of PTB. And no correlation indicators were excluded. The standardized coefficient and variance inflation factor (*VIF)* were calculated by linear regression. When *VIF*>=10, it indicated that there was a multi-collinearity problem between variables. The multi-collinearity index was removed, and the optimal combination was obtained after several fitting experiments.

### Geographically and temporally weighted regression model

The traditional global regression model OLS cannot reflect the spatial heterogeneity of different regional coefficients, nor can it effectively excavate important local features between explanatory variables and explained quantities. GWR can only be used for cross-sectional data and cannot consider the time factor [9]. In order to consider the information of time and space, Huang Bo proposed the GTWR model [10]. The GTWR model determined the shadow specific gravity of other sample points on the regression sample points by constructing the space–time weight matrix. Therefore, the space–time weight matrix played a core role in the calculation process of the GTWR model. Its form was a diagonal matrix, and the elements in the matrix were determined by three factors, such as space bandwidth, kernel function, and distance calculation formula. This paper was based on adaptive bandwidth, Gaussian kernel function, and Euclidean distance and determined by Akaike Information Criterion corrected (*AICc*). It was a useful tool when comparing models with different explanatory variables, as long as it was applied to the same dependent variable. If the *AICc* values of two models differed by more than 3, the model with the lower *AICc* value was generally considered superior.

## Results

### Principle component analysis

The result of PCA was illustrated in Table 2. PCA of *KMO* test values was greater than 0.06, *P* < 0.05.

**Table 2.** Normalized values for the principal component scores

No.	Province	Time	Medicine and health	Transportation	Environment	Economy	Disease	Educational quality
1	Beijing	2004	0.084	0.074	0.091	0.234	0.022	0.487
2	Tianjin	2004	0.037	0.012	0.094	0.148	0.006	0.225
3	Hebei	2004	0.174	0.305	0.684	0.062	0.001	0.272
4	Shanxi	2004	0.107	0.119	0.665	0.062	0.005	0.178
…	…	…	…	…	…	…	…	…
555	Gansu	2021	0.266	0.172	0.136	0.297	0.093	0.277
556	Qinghai	2021	0.074	0.057	0.032	0.349	0.149	0.079
557	Ningxia	2021	0.058	0.044	0.055	0.375	0.068	0.136
558	Xinjiang	2021	0.258	0.242	0.246	0.36	0.253	0.280

### Spearman’s correlation test

The Spearman correlation test showed that six macro-factors and incidence of PTB were correlated. The *P* values between medicine and health, transportation, environment, economy, disease, and educational quality and the incidence of PTB are <0.001, <0.001, 0.004, <0.001, 0.003, and <0.001, respectively.

### Multi-collinearity test

The result of multi-collinearity test (Table 3) showed that each variable’s *VIF* was less than 10, and the variable did not have a collinearity problem.

**Table 3.** The *VIF* index of macro-factors of incidence of PTB

	Standardized coefficient	VIF
Medicine and health	−0.187	7.742
Transportation	0.358	7.211
Environment	−0.073	2.209
Economy	−0.297	2.62
Disease	0.208	1.314
Educational quality	−0.472	6.034

### The comparisons of OLS, GWR, and GTWR models

Based on the fitting effect of different models, the result of comparisonswas shown in Table 4. The GTWR model had the highest *R^2^* value and the lowest *AICc* value compared with the other two models, demonstrating that the GTWR model outperformed OLS and GWR for determining the relationship between incidence of PTB and six macro-factors.

**Table 4.** Values of *R^2^* and *AICc* of OLS, GWR, and GTWR models

	OLS	GWR	GTWR
*R^2^*	0.400696	0.874037	0.88975
*AICc*	5342.206501	4576.42	4534.51

### Regression coefficient characteristics of space and time

**Figure 1.**
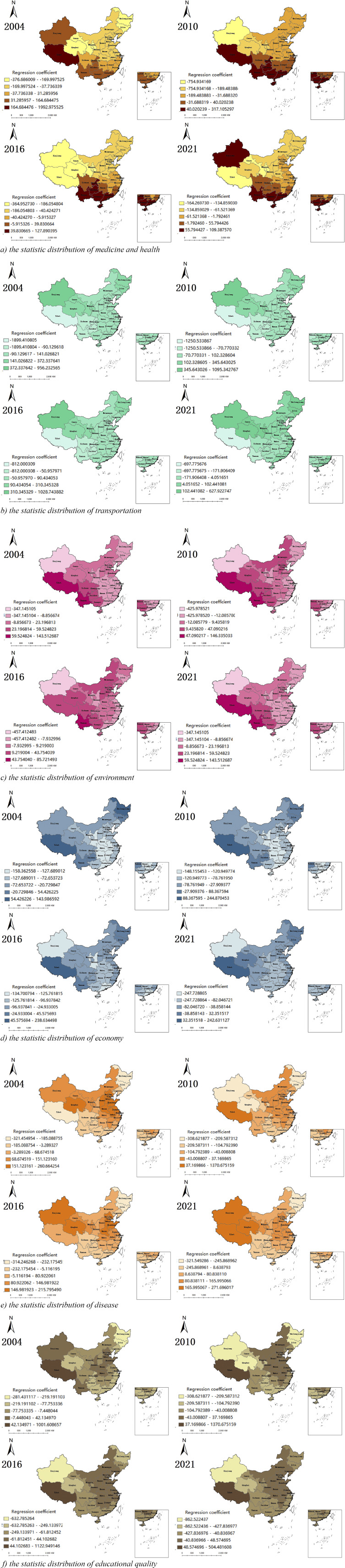
2004, 2010, 2016, and 2021 GTWR regression coefficient distribution.

The estimated results of the GTWR model was shown in Table 5. Medicine and health, transportation, environment, economy, disease, and educational quality of the regression coefficient of the average were -30.99, 38.77, -6.35, -65.54, 54.19, and -37.73, respectively. The influence of various macro-factors on PTB was economy, disease, transportation, educational quality, medicine and health, and environment. The macro-factor regression coefficient after visual situation was shown in Figure 1.

**Table 5.** Estimates of the GTWR model

	Mean	Min	Lower quartile	Median	Upper quartile	Max
Intercept	101.95	41	80.80	97.72	123.50	285
Medicine and health	−30.99	−1 021	−63.37	−38.51	30.13	1 993
Transportation	38.77	−1 899	−1.37	25.17	55.75	1 106
Environment	−6.35	−457	−12.67	−1.24	15.60	222
Economy	−65.54	−248	−110.72	−79.10	−48.75	248
Disease	54.19	−322	−25.71	62.82	146.60	272
Educational quality	−37.73	−863	−119.67	−45.4	10.03	1 377

## Discussion

To this day, China still has one of the highest PTB burdens. The analysis on the influencing factors of PTB was mostly focused on a single province or city and other local areas. This paper conducted modelling analysis on the incidence and influencing factors of PTB at the provincial level throughout the country from 2004 to 2021 and found that compared with the traditional OLS model and GWR model the overall fitting effect of the GTWR model was better.

The results of the GTWR model showed that different regions were affected by different macro-factors to different degrees; the incidence of PTB in China was spatially heterogeneous in different provinces and cities. From 2004 to 2021, the principal factors affecting the incidence of PTB in China were economy and disease. In most areas of China, economy was negatively correlated with the incidence of PTB, while disease was positively correlated with the incidence of PTB. Among them, Xinjiang Uygur Autonomous Region [11–14] was highly affected by economic and disease factors, and the influence degree was increasing. This may be related to the shortage of local medical expenditure in areas with relatively backward economic development [15–18], resulting in delayed diagnosis and treatment. The secondary factors affecting the incidence of PTB in China were transportation and educational quality. The transportation factors had a significant difference in the incidence of PTB in different areas, while the educational quality factors had a negative correlation with the incidence of PTB. We found that in Northwest, Northeast, and North China, traffic indicators were positively correlated with the incidence of PTB, possibly because population flow increased the risk of spreading PTB bacilli, while in other regions with negative correlation, the most significant impact was in Southwest China, which may be related to the development of local transportation driving the development of tourism and thus promoting the development of economy. The awareness of health screening and regular physical examination may be poor in areas with low education level, and early detection, diagnosis, and treatment of diseases cannot be achieved. Educational status and health awareness among TB patients can influence their lifestyles in order to improve their living environments to prevent the spread of infectious disease [19]. Health education interventions and efforts were needed to strengthen precise information dissemination to promote knowledge, attitudes, and practices regarding TB among patients and non-patients at primary healthcare facility. In addition, medical and health care and the environment also had a certain impact on the national incidence of PTB; the lower the medical and health care and the more serious the environmental pollution, the higher the incidence of PTB [20–22]. This suggested that we should strengthen the medical and health infrastructure, improve the medical service system, increase the number of designated medical institutions for PTB, and do a good job in environmental protection.

The incidence of PTB in China had spatial–temporal heterogeneity and was affected by the economy, disease, transportation, educational quality, medical and health care, environment, etc. It was characterized by a large difference between the north and the south in space and between the east and the west. In terms of time, the influencing degree of each factor was also different. In addition, this study also had some shortcomings, such as taking provincial administrative regions as the unit and failing to be accurate for prefecture-level cities. However, this study had a long-time span and a wide range of influencing factors, which can accurately reflect the temporal and spatial trend of the PTB epidemic.

Therefore, the following suggestions are put forward: First, Qinghai, Tibet, Gansu, and other regions should speed up economic construction, strengthen medical and health infrastructure construction, improve the medical service system, improve the diagnosis rate of PTB, and reduce under-reporting. Second, they should strengthen the construction of transportation in rural areas, expand the construction of roads and railways, and make it easier for residents in rural and remote areas to see a doctor in a timely manner. At the same time, for areas with developed traffic, the floating population should be checked in time to prevent the flow of people from being too dense. Third, in southwest and North China, China will expand publicity, raise awareness of household waste classification and treatment, improve urban greening, reduce the emission of harmful substances in waste gas, and improve air quality. In areas with high incidence of PTB, health publicity and education on infectious diseases should be carried out in communities and schools, at the same time, make the integration of knowledge, belief, and practice. In particular, ethnic minority gathering areas should strengthen publicity and improve awareness of PTB.

## Conclusions

The affecting factors of the GTWR model on the incidence of PTB in China were comparatively reasonable, which reflected that the incidence of PTB had spatial–temporal heterogeneity. The incidence of PTB was mainly affected by six macro-factors, namely medicine and health, transportation, environment, economy, disease, and educational quality.

## Data Availability

The extracted data are available from the request from the corresponding author.
